# A systematic review of the efficacy of self-management programs for increasing physical activity in community-dwelling adults with acquired brain injury (ABI)

**DOI:** 10.1186/s13643-015-0039-x

**Published:** 2015-04-19

**Authors:** Taryn M Jones, Catherine M Dean, Julia M Hush, Blake F Dear, Nickolai Titov

**Affiliations:** 1 Department of Health Professions, Macquarie University, Ground Floor, 75 Talavera Rd, Sydney, NSW 2109 Australia; 2 Department of Psychology, Centre for Emotional Health, Building C3A, Level 7, Macquarie University, Sydney, 2109 Australia; 3 Centre for Physical Health, Macquarie University, Ground Floor, 75 Talavera Rd, Sydney, 2109 Australia

**Keywords:** Management, Exercise, Trauma, Cerebrovascular accident, Remote delivery, Internet

## Abstract

**Background:**

Individuals living with acquired brain injury, typically caused by stroke or trauma, are far less likely to achieve recommended levels of physical activity for optimal health and well-being. With a growing number of people living with chronic disease and disability globally, self-management programs are seen as integral to the management of these conditions and the prevention of secondary health conditions. However, to date, there has been no systematic review of the literature examining the efficacy of self-management programs specifically on physical activity in individuals with acquired brain injury, whether delivered face-to-face or remotely. Therefore, the purpose of this review is to evaluate the efficacy of self-management programs in increasing physical activity levels in adults living in the community following acquired brain injury. The efficacy of remote versus face-to-face delivery was also examined.

**Methods:**

A systematic review of the literature was conducted. Electronic databases were searched. Two independent reviewers screened all studies for eligibility, assessed risk of bias, and extracted relevant data.

**Results:**

Five studies met the inclusion criteria for this review. Studies were widely heterogeneous with respect to program content and delivery characteristics and outcomes, although all programs utilized behavioral change principles. Four of the five studies examined interventions in which physical activity was a component of a multifaceted intervention, where the depth to which physical activity specific content was covered, and the extent to which skills were taught and practiced, could not be clearly established. Three studies showed favorable physical activity outcomes following self-management interventions for stroke; however, risk of bias was high, and overall efficacy remains unclear. Although not used in isolation from face-to-face delivery, remote delivery via telephone was the predominant form of delivery in two studies with support for its inclusion in self-management programs for individuals following stroke.

**Conclusions:**

The efficacy of self-management programs in increasing physical activity levels in community-dwelling adults following acquired brain injury (ABI) is still unknown. Research into the efficacy of self-management programs specifically aimed at improving physical activity in adults living in the community following acquired brain injury is needed. The efficacy of remote delivery methods also warrants further investigation.

**Systematic review registration:**

PROSPERO CRD42013006748

**Electronic supplementary material:**

The online version of this article (doi:10.1186/s13643-015-0039-x) contains supplementary material, which is available to authorized users.

## Background

Acquired brain injury (ABI) refers to any damage to the brain that occurs after birth with common causes including stroke or trauma [1]. ABI is a significant public health issue globally. Stroke is one of the greatest causes of disease burden globally [2] and is one of the main non-communicable diseases of public health importance [3], while traumatic brain injuries are the leading cause of disability in children and young adults globally [3].

Individuals with ABI often have more complex disabilities than other groups with disability [1] and often face many barriers in increasing their levels of physical activity, such as mobility impairments, fear, pain, financial costs, transport difficulties, and limited local specialist services [4-9]. Physical activity interventions are effective in improving physical, psychosocial, and cognitive status; however, maintaining these improvements once intervention ceases is challenging, and physical activity participation levels after ABI remain low [5,10-16].

Physical inactivity both causes and accelerates chronic diseases, such as cardiovascular disease, diabetes, and cancer [17], with individuals with ABI at elevated risk [1]. In fact, the World Health Organization (WHO) report that almost a third of all strokes occur in those who have previously had a stroke [3]. Despite this, ABI is often a lower priority for research and services than conditions with a similar, or lower, public health priority [2] and there is a significant lack of physical activity promotion programs targeting those with ABI [18,19].

The WHO has argued for nations to do more to prevent chronic disease [20], particularly through the use of strategies to increase physical activity [21]. Self-management is seen as integral to optimal chronic disease prevention and management [22]. Given that physical inactivity is a significant modifiable risk factor for chronic diseases, such as stroke [17,23], increasing the self-management of physical activity specifically in individuals with ABI appears crucial for the long-term prevention of further morbidity and mortality.

Self-management ultimately reflects an individual’s responsibility for the day-to-day management of their disease including decisions regarding engagement in healthy behaviors [24]. The most promising way of supporting self-management is to empower and activate people, primarily through the expansion of skills, such as problem-solving and decision-making, therefore building self-efficacy to alter long-term behaviors [24,25]. There is considerable evidence that self-management programs result in better long-term outcomes for people with chronic diseases [26-29], including programs for individuals with ABI, specifically stroke [30,31]. Despite this, many people with ABI do not receive and cannot access self-management programs. For example, in the National Stroke Audit undertaken in Australia in 2012, only 25% of stroke survivors were informed about self-management programs, a decline from 40% in 2008 [32].

Implementation of self-management programs may be enhanced through the use of innovative modes of remote program delivery. Compared with face-to-face delivery, remote delivery modes, such as telephone and the Internet, may increase accessibility for those who face multiple barriers to accessing optimal health care [33], such as cost, mobility restrictions, or service availability in rural or remote regions. Delivery of self-management programs via the Internet has been used with success in a variety of populations, such as chronic pain [34], anxiety and depression [35-37], post-traumatic stress disorder [38], arthritis [39], and cerebral palsy [40]. The potential for remote-based delivery methods to be utilized to increase physical activity has also been highlighted by Foster and colleagues in a recent Cochrane review [41]. However, to date, there has been no systematic review of the literature examining the efficacy of self-management programs specifically on physical activity in individuals with ABI, whether delivered face-to-face or remotely. Therefore, the objectives of this systematic review were to address the following questions:

1. How effective are self-management programs in improving physical activity in community-dwelling adults with ABI?
2. How effective and acceptable is remote delivery of self-management programs aimed at improving physical activity in community-dwelling adults with ABI?
3. Which features of self-management programs for community-dwelling adults with ABI are associated with the best clinical outcomes and client satisfaction?

## Methods

### Study registration

The systematic review has been conducted and reported using the Preferred Reporting Items for Systematic Reviews and Meta-Analyses (PRISMA) statement guidelines [42]. The Cochrane Collaboration guidelines for reviewing interventions were also closely followed [43]. The protocol of this systematic review has been registered on PROSPERO 2013 (registration number: CRD42013006748) [44] and has been published [45].

### Search strategy

We conducted an extensive search of the literature for articles indexed on MEDLINE, EMBASE, CINAHL, PsycINFO, AMED, Cochrane Central Register of Controlled Trials (CENTRAL), PEDro and Science Citation Index Expanded (SCI-EXPANDED) databases from their inception to December 2014. We developed a search strategy in MEDLINE using the following steps: (1) development of keywords by examining relevant key terms used in existing systematic reviews, (2) a thorough examination of the MeSH Database, and (3) expert guidance and review by a specialist librarian. Finally, the search strategy was trialed and refined in order to ensure it was the most effective strategy for this review (Additional file 1). This strategy was then customized for differences in indexing across other databases [45] published to allow for replication [45]. We also screened the reference lists of relevant reviews to identify further studies for potential inclusion in this review. Non-English language studies were also considered for inclusion, where a translation could be made available.

### Eligibility criteria

Our eligibility criteria were defined *a priori* and are outlined in Table 1. ABI was defined as damage to brain occurring after birth. However, for the purpose of this review, studies examining individuals with degenerative ABI (for example Parkinson’s disease or multiple sclerosis), cerebral palsy, developmental delay, fetal alcohol spectrum disorder (FASD), concussion, or transient ischaemic attacks (TIA) were not included. There was no limit based on time since injury. In studies where it was unclear that participants met our inclusion criteria, we contacted the study author for verification. We excluded any studies where verification could not be made by the authors.

**Table 1:** **Inclusion criteria**

	Inclusion
Study design	Randomized controlled trial (RCT)
	Quasi-randomized controlled trial (QRCT) - for example, allocation by date of birth, location, medical record number
Participants	Adults (18 years and over)
	Non-degenerative acquired brain injury (ABI)
	Currently living in the community
	Are not undergoing significant medical or surgical intervention
Intervention	Self-management program which:
	Includes at least one of the following components: problem-solving, goal-setting, decision-making, self-monitoring, coping strategies, or another approach to facilitate behavior change;
	Has at least a component of the program focusing on increasing physical activity.
Outcomes	Must include at least one of the following:
	A measure of physical activity: either from a physical activity monitoring device (for example, accelerometer, pedometer) or a self-report measure;
	And/or
	A study outcome associated specifically with physical activity, for example, physical activity self-efficacy, physical self-concept, or stages of change in relation to physical activity.

### Identification of relevant studies

Two authors (TMJ, CMD) independently assessed the titles and abstracts of all records identified from the searches of the electronic databases. Records identified as not meeting the eligibility criteria were excluded. The full text of the remaining studies was obtained and reviewed for eligibility independently by the same two authors. In one case, an independent translation from Korean to English was required in order to assess eligibility. At each stage of the process, records were marked ‘accept’, ‘reject’, or ‘unsure’. Those records marked ‘unsure’, or where disagreements between reviewers arose, discussion between the reviewers was undertaken in order to reach consensus.

### Data extraction

Data from included studies were extracted independently by two reviewers (TMJ, CMD) using a standardized data extraction form. Data were extracted for all available time points on the outcome measures that were defined *a priori* as per our protocol [45]. We also recorded any adverse outcomes that were reported in the studies included in this review.

### Risk of bias assessment

Two reviewers (TMJ, CMD) independently assessed the risk of bias for each included study using *The Cochrane Collaboration’s tool for assessing bias* [46]. A summary of risk of bias across all studies within each domain was also performed.

### Data synthesis

A meta-analysis was not possible due to significant heterogeneity of the outcome measures utilized in each of the studies. Instead, a detailed summary of the results from the individual studies was collated into a table, and a systematic narrative synthesis was conducted. A comparison of remote-delivery methods with traditional face-to-face delivery methods was also not possible because all studies included in the review included a face-to-face delivery mode for at least some portion of their program.

## Results

### Results of the search

**Figure 1:**
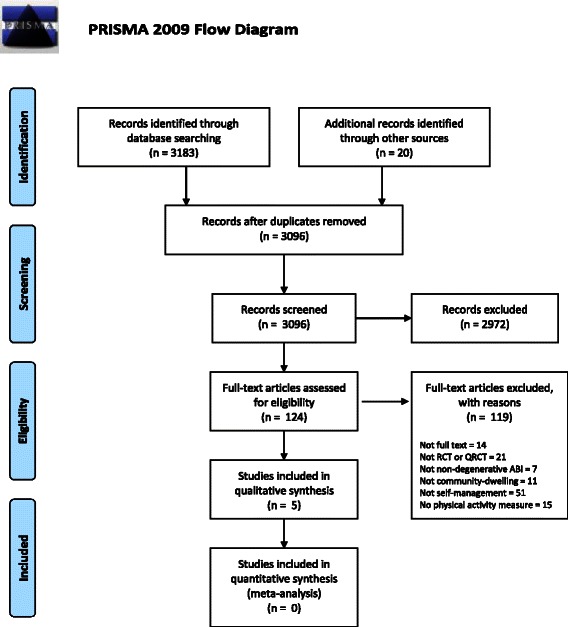
Study flow diagram.

Our search of electronic databases generated 3,654 references. An additional 20 references were obtained from handsearching the reference lists of nine systematic reviews identified from the electronic searches [31,47-54]. Following duplicate removal and screening of titles and abstracts, 124 full-text articles were assessed for eligibility. Assessment resulted in 119 references being excluded with reasons outlined in Figure 1. Five studies met the eligibility criteria and were included in this review [55-59].

### Details of included studies

Details of the included studies are reported in Table 2. Four of these studies were published in English. The fifth study was published in Korean [58], and an independent translation was obtained. Two studies were performed in the USA [55,56], one in Australia [57], one in Korea [58], and one in Hong Kong [59]. The interventions examined in each study varied in regard to both content and delivery characteristics. The studies also vary greatly in regard to outcome measures used.

**Table 2:** **Summary of included studies**

Study (year, country, study design)	Type of ABI	Participants	Intervention			Control	Follow-up assessments/
			Content	Delivery characteristics	Theoretical model		Drop outs/
							Sample size analyzed
Brenner *et al*. (2012, USA, RCT)	TBI	Sample size: *n* = 74	**‘**Health and Wellness therapy group’: program provided information to facilitate health promotion while emphasizing self-assessment to help participants to set individualized goals, problem-solve to reduce barriers, and strategies to enhance self-efficacy. Program aimed to take advantage of the group process, as well as encouraging participants to involve an identified resource person to assist in self-assessment and behavior change.	Duration: 12 × 1.5 h sessions; 1 session/week for 12 weeks	TTM	Wait-list control	Follow-up: 3 months and 6 months
		IG = 37; CG = 37			SCT		
		Gender:					
		Male: IG = 29 (78.4%)					
		CG = 32 (86.5%)					Drop outs: *n* = 9
		Female: IG = 8 (21.6%)					IG: *n* = 7; CG: *n* = 2
				Delivery mode: face-to-face group sessions with workbook			
		CG = 5 (13.5%)					
							Sample analyzed: *n* = 65
		Mean age (years):					
							IG: *n* = 30; CG: *n* = 35
		IG = 43.46 (SD 16.00);		Facilitators: social worker, speech pathologist, physical therapist, and nurse who rotated in groups of 2			
		CG = 44.14 (SD 14.97)					
		Mean (SD) time since ABI (years):					
		IG = 11.74 (13.80);	Physical activity specific content: Two sessions (sessions 5 and 6) focus on fitness self-assessment, getting started with physical exercise, measuring resting heart rate, benefits of exercise.				
		CG = 12.50 (13.75)					
Damush *et al*. (2011, USA	Stroke	Sample size: *n* = 66	‘Stroke self-management program’: The sessions followed a standardized manual based on the CDSMP with a focus on enhancing self-efficacy to manage symptoms and foster behavior change. Techniques employed included goal setting and behavioral contracting. Telephone follow-up focused on reinforcing, monitoring, and adjusting the goals and self-management strategies.	Duration: 6 sessions over a 3-month period (3 face-to-face and 3 via telephone) as well as biweekly telephone follow-up. Average session length was 20 min.	SCT (specifically self-efficacy)	Written patient educational materials on stroke warning signs and pamphlets from the American Stroke Association on prevention of secondary strokes. Telephone calls were also made by the case manager on the same schedule as IG to discuss how participant felt that day.	Follow-up: 3 months and
		Gender:					
		Male: IG = 30 (100%)					
RCT)							
		CG = 32 (97.0%)					
		Female: IG = 0 (0%)					6 months
		CG = 1 (3.0%)					Drop outs: *n* = 3
							No info regarding groups
		Mean age (years):					Sample analyzed: *n* = 63
		IG = 67.3 (SD 12.4);					
							IG: *n* = 30; CG: *n* = 33
		CG = 64.0 (SD 8.4)		Delivery mode: face-to-face and telephone with standardized manual			
		Time since ABI: participants identified during hospital admission for ischemic stroke.	Physical activity specific content: 2 topics out of 24 focused on physical activity specifically - ‘Getting Active at Home’ and ‘Walking for Health’. An additional topic on rehabilitation included discussion on following prescribed exercises at home.				
				Facilitators: a nurse, a physician assistant, and a master’s level social scientist			
Gill and Sullivan (2011, Australia QRCT)	Stroke	Sample size: *n* = 26	‘Stay Active and Stop Stroke (SASS)’: Intervention targets exercise beliefs with didactic instruction and group-based activities. Session 1 aimed to increase stroke knowledge and highlight risk factors. Session 2 aimed to facilitate a change in beliefs. Session 3 intended to strengthen motivation by illustrating decisional balance processes. Participants identified personal barriers to increasing physical activity, generated possible solutions, and prepared personal activity goals.	Duration: 3 × 1 h sessions, 1/week for 3 weeks.	eHBM	No intervention	Follow-up: 3 weeks
		IG: *n* = 14; CG: *n* = 8					
					TTM		Drop outs: *n* = 0
		Gender:					IG: *n* = 0; CG: *n* = 0
		Male: IG = 5 (35.7%)					
		CG = 6 (75%)		Delivery mode: face-to-face group sessions with manual			
		Female: IG = 9 (64.3%)					
		CG = 2 (25%)					Sample analyzed: *n* = 26
							IG: *n* = 14; CG: *n* = 8
		Mean age (years):		Facilitators: psychology students			
		IG = 60.21 (SD 7.74);					
		CG = 67.75 (SD 19.30)					
		Time since ABI:					
		<12 months: IG: *n* = 2; CG: *n* = 1;					
			Physical activity specific content: Whole program focused on exercise.				
		1 to 5 years: IG: *n* = 7; CG: *n* = 4;					
		>5 years: IG: *n* = 5; CG: *n* = 3					
Kim and Kim	Stroke	Sample size: *n* = 61	‘Lifestyle modification coaching program’: Aimed to modify lifestyle to prevent secondary stroke, particularly through reduction in physiological parameters, such as blood pressure, blood lipids, and body fat. Program focused on education regarding stroke risk factors and acknowledgement of necessity for lifestyle modification, as well the setting up and attainment of individual goals.	Duration: 8 weeks	None specified	Control received the 1 × face-face session but no ongoing telephone coaching.	Follow-up: 8 weeks
(2013, Korea		IG: *n* = 32; CG: *n* = 29					
				Delivery mode: Initial session was face-to-face, then telephone (1× week for 8 weeks)			Drop outs: n = 12
							IG: n = 5; CG: n = 7
QRCT)		Gender:					
		Male: IG = 19 (59.4%)					
		CG = 19 (65.5%)					
		Female: IG = 13(40.6%)					Sample analyzed: *n* = 61
		CG = 10 (34.5%)					
							IG: *n* = 32; CG: *n* = 29
				Facilitators: not specified			
		Mean age (years):					
		IG: 67.41 (8.46)					
		CG: 66.71 (9.40)	Physical activity specific content: Participants were classified according to their baseline level of activity and encouraged to acknowledge their current level of activity. Subjects educated about optimum levels of exercise to prevent stroke recurrence, and assisted to set goals and keep records on exercise performed. The researcher checked if reasonable exercise was being done, offered encouragement, and gave support to identify and overcome barriers.				
		Median (range) time since ABI (months): IG: 24 (2 to 124) CG: 36 (2 to 188)					
Sit *et al*. (2007, Hong Kong, QRCT)	Stroke	Sample size: *n* = 190	‘Community-based stroke prevention program’: Focus was on improving knowledge about stroke, improving self-monitoring of health and maintenance of behavioral changes when adopting a healthy lifestyle. Participants selected the risk behavior on which they wanted to focus, addressing them one at a time, setting short-term practical goals, practicing learnt skills, and implementing action plans.	Duration: 8 × 2 h sessions held 1/week for 8 weeks.	None specified	Conventional medical treatment and health promotion pamphlets on stroke and stroke prevention.	Follow-up: 1 week following intervention and 3 months
		IG: *n* = 107; CG: *n* = 83					
		Gender:					
		Male: IG = 55 (51.4%)		Delivery mode: face-to-face group sessions with 10 to 12 participants.			
		CG = 50 (60.2%)					Drop outs: *n* = 44
							IG: *n* = 28; CG: *n* = 16
		Female: IG = 52 (48.6%)					
		CG = 33 (39.80%)					
							Sample analyzed:
							*n* = 190
		Mean age (years):		Facilitators: experienced community nurses.			IG: *n* = 107; CG: *n* = 83
		IG = 62.83 (SD 10.25);					
		CG = 64.02 (SD 12.03)					
		Time since ABI: not specified					
			Physical activity specific content: Participants were given log sheets and pedometers to track goal achievement. Physical activity was focused on in session 7: ‘Establishing regular exercise habit’.				

IG = Intervention group; CG = Control group; TTM = Transtheoretical Model; SCT = Social Cognitive Theory; CDSMP = Chronic Disease Self-Management Program; eHBM = expanded Health Beliefs Model.

#### Demographic characteristics

Demographic details of study participants are outlined in Table 2. Four of the studies examined participants following stroke [56-59], while one studied participants with traumatic brain injury (TBI) [55]. Most studies included in this review had relatively small sample sizes, although one study had 190 participants [59]. The mean age of all stroke participants (*n* = 336) was 64.42 (SD = 10.81) years, while the TBI participants (*n* = 74) had a mean age of 43.83 (SD = 15.34) years. A measure of severity of ABI was reported in two of the five studies [55,56]. Four out of the five studies [55,56,58,59] reported eligibility criteria that required cognitive and communication skills to be adequate for participation in a self-management program; however, assessment of this criterion differed in each study.

#### Intervention content

The content of the intervention programs applied in each of the studies is also summarized in Table 2. The extent to which physical activity was specifically addressed and targeted differed between each program. In four of the five studies, physical activity, or exercise, was included as a subtopic within a larger program covering numerous aspects of self-management skills following acquired brain injury, such as diet modification, stress management, and medication compliance [55,56,58,59]. The intervention evaluated by Gill and Sullivan [57] was the only one that focused solely on exercise, with an intervention designed to boost exercise beliefs and motivation.

Three of the five studies applied theoretical models of health behavior change in developing their intervention content [55-57]. Theories utilized included Bandura’s social cognitive theory (SCT) [60,61], Prochaska’s transtheoretical model (TTM) [62], and the expanded health beliefs model (eHBM) [63]. The remaining two studies [58,59] utilized similar behavior change principles in an educational framework, with a focus on building knowledge regarding current stroke management and stroke risk factors, individual goal setting, and self-monitoring.

#### Delivery characteristics

The delivery characteristics of the intervention programs are outlined in Table 2. Most of the interventions were delivered during an 8- to 12-week time frame. All the interventions included at least some element of face-to-face delivery; however, two studies delivered the majority of their intervention remotely via telephone [56,58]. The three studies that utilized only face-to-face delivery all did this via group sessions [55,57,59]. Standardized manuals or workbooks to assist in the delivery of the intervention were utilized in three studies [55-57]. All interventions were facilitated by health professionals, including a multidisciplinary team facilitating sessions in two of the five studies [55,56]. Nurses were most commonly engaged in the role of facilitator [55,56,59].

#### Outcome measures

Each of the included studies reported on a different set of outcome measures to examine physical activity, as summarized in Table 3. Three studies measured physical activity specifically [56,58,59]. Damush and colleagues recorded self-reported time spent in aerobic activity each week [56]. Kim and Kim recorded weekly metabolic equivalent of task (MET) minutes by using self-reported information from a translated version of the International Physical Activity Questionnaire (IPAQ) [58]. Sit and colleagues utilized an exercise subscale modified from Lorig [64] and reported physical activity data as the proportion of the group that participated in walking exercise [59]. The remaining two studies utilized different validated questionnaires regarding physical activity, including the Health Promoting Lifestyle Profile - II (HPLP-II) Physical Activity subscale [55], the Self-Rated Abilities for Health Practices (SRAHP) Exercise subscale [55], and the Cerebrovascular Attitudes and Beliefs Scale - Revised (CABS-R) Exercise subscale [57].

**Table 3:** **Summary of results**

Study	Measure used	Results
Brenner *et al*. [55]	Physical activity	Raw data: No raw data reported.
	measure: HPLP-II Physical Activity Subscale	Group comparisons: Data reported as time-by-treatment interaction (*P*) - no significant differences between the IG and CG in regard to HPLP-II (Physical Activity) (*P* = 0.2375) or SRAHP (Exercise) (*P* = 0.3661).
	SRAHP Physical Activity & Exercise domain	
		Both these values reached significance (*P* = 0.0216 and *P* = 0.0001 respectively); however, the authors state differences are due to time, not treatment.
	Other measures: Participation Assessment with Recombined Tools-Objective (PART-O)	
	Diener Satisfaction with Life Scale	
Damush *et al*. [8]	Physical activity measure: Self-reported time spent in aerobic activity (min/week)	Raw data [1] :
		IG: Baseline = 78.5 min/week; 3 months = mean increase of 47.6 min/week. CG: Baseline = 107.4 min/week; 3 months = mean decrease of 3 min/week.
		Between-group comparison: 3 months: *t*_(51)_ = 1.18, *P* ≤0.13, effect size = −0.43; 6 months: *P* ≤ 0.50, effect size = −0.19
	Other measures: Stroke-Specific Health-Related Quality of Life (SSQOL)	
		Not all data supplied. At baseline, the IG had significantly lower (worse) scores for several SSQOL scales including mobility, thinking, energy, and work, as well as the total overall score. For both the subscales of Family Roles and Social Roles, the IG improved at 3 months, while the CG declined with differences between the groups reaching significance (*P* ≤ 0.01 and *P* ≤ 0.06, respectively).
Gill and Sullivan [57]	Physical activity measure:	Raw data: Mean (SD) self-ratings.
	Cerebrovascular Attitudes and Beliefs Scale-Revised (CABS-R) Exercise subscale	Barriers: IG: T1 = 2.19(0.76), T2 = 2.35(0.67); CG: T1 = 2.22(0.49), T2 = 2.27(0.74)
		Benefits: IG: T1 = 3.90(0.73), T2 = 3.94(0.46); CG: T1 = 3.59(0.67), T2 = 3.53(0.60)
		Susceptibility: IG: T1 = 3.62(0.86), T2 = 3.69(0.60); CG: T1 = 2.42(0.94), T2 = 2.92(0.61).
		Seriousness: IG: T1 = 4.18(1.05), T2 = 4.26(0.76); CG: T1 = 3.71(1.38), T2 = 3.50(1.41)
	SOEQ (stages of change, 1 item)	Self-efficacy: IG: T1 = 3.31(0.90), T2 = 3.77(0.53); CG: T1 = 3.13(1.09), T2 = 3.25(1.00)
		Subjective norms: IG: T1 = 4.27(0.53), T2 = 4.08(0.53); CG: T1 = 4.06(0.18), T2 = 4.06(0.18)
		Within-group comparison: IG showed a significant increase in self-efficacy from baseline to follow up (*F*(1, 11) = 7.33; *P* = 0.02).
		Between-group comparison: IG reported significantly higher perceptions of susceptibility than CG at both time points (baseline *P* = 0.007 and 3 weeks *P* = 0.010). No other differences were found.
		The IG had a small movement of 14.3% (*n* = 2) from the preparation stage to the active stage at 3 weeks which was not seen in the CG on SOEQ categorical data. No other changes were found.
Kim and Kim [58]	Physical activity measure: Physical activity: MET minutes/week	Raw data: Median (range)
		IG: Baseline = 462.0 (0.0 to 3,942.0), 8 weeks = 1,365.5 (132.0 to 4,158.0)
		CG: Baseline = 984.0 (0.0 to 6,906.6), 8 weeks = 990.0 (0.0 to 25,638.0)
	Other measures: General Self-Efficacy Scale	
		Within-group comparison: IG showed significant increase in weekly MET minutes at 8 weeks with a difference in median between baseline and 8 weeks to be 601.5 MET min/week (range −2,628.0 to 3696.0; *T* = 149; *P* = 0.001); CG showed a non-significant change with a difference in median to be 133.0 MET min/week (range −4,976.0 to 25,638.0; *T* = −30.50; *P* = 0.474).
		Between-group comparison: Difference in change over 8 weeks was significantly different between groups in favor of IG (*T* = 692.50; *P* = 0.002).
		No significant differences found within groups or between groups in general self-efficacy.
Sit *et al.* [59]	Physical activity measure: Participation in walking exercise	Raw data: Percentages reported; T0 = baseline, T1 = postone week, T2 = 3 months
		IG: T0 = 78.9%, T1 = 78.9%, T2 = 77.1%
		CG: T0 = 72.3%, T1 = 63.9%, T2 = 55.4%
		Within-group comparison: At 3 months: IG Q = 0.051; *P* = 0.975, CG Q = 7.697; *P* = 0.021
		Between-group comparison: At 3 months, there was a significant difference between groups in favor of the IG (*P* < 0.001)

Data from Damush *et al*. (2011) included 6 month data that reported a mean increase in the IG of 24.4 min/week and a mean increase in the CG of 4 min/week, with a between-group comparison of *t*_(52)_ = −0.69, *P* ≤ 0.50, effect size = −0.19; however, this data was not reported in this table as it was unclear as to whether these increases were from baseline or from 3 months.

In addition to these specific physical activity measures, a wide variety of secondary outcome measures were used by the authors to examine other factors associated with self-management of acquired brain injury, such as self-efficacy for communicating with physicians [56] or smoking and alcohol behavior [58]. We extracted data only from those measures that were aligned with the secondary outcome measures outlined in our protocol [45]. These results are summarized in Table 3. No studies employed outcome measures to examine participant satisfaction or program cost-effectiveness. Adverse events were also not reported in any of the studies included in this review.

### Risk of bias of included studies

**Figure 2:**
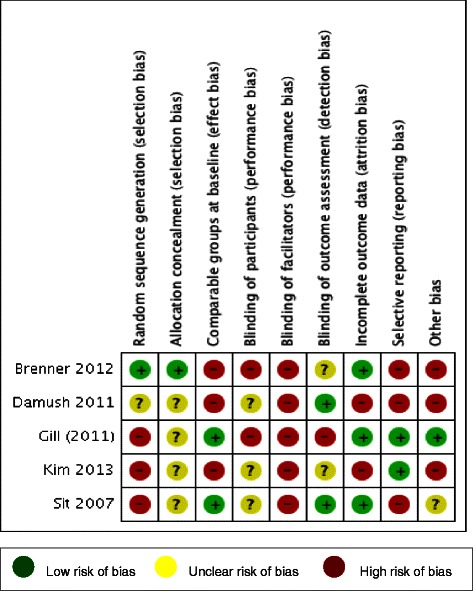
Risk of bias summary - review authors’ judgements about each risk of bias item for each included study.

**Figure 3:**
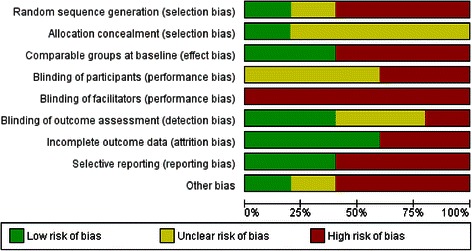
Risk of bias graph - review authors’ judgements about each risk of bias item presented as percentages across all included studies.

Risk of bias for each study is summarized in Figure 2, with a summary of each risk of bias item detailed in Figure 3. Overall, risk of bias was generally high across all parameters. Four of the five studies are at high risk of selection bias with only one study providing clear information regarding adequate random sequence generation and allocation concealment [55]. Blinding of facilitators is impossible in these types of studies and blinding of participants is also challenging, but none of the included studies demonstrated clarity regarding blinding of participants [56-59]. This is particularly pertinent in these studies where data was collected through self-report measures. As a result, all studies were considered to be at high risk of performance bias. Three studies were considered to be at high risk of reporting bias with data not fully presented and/or difficult to analyze [55,56,59]. Other potential sources of bias arose due to differences in groups at baseline regarding physical activity measures, issues regarding the delivery and monitoring of control interventions, and the use of *post hoc* statistical analysis techniques [55,56,58].

### Effects of interventions

#### Efficacy in improving physical activity

A summary of results is displayed in Table 3. As stated previously, a meta-analysis was not possible due to the significant variability in outcome measures utilized in each study. Therefore, a pooled estimate of efficacy cannot be established at this stage. However, in one study of stroke survivors, there is evidence that an 8-week lifestyle modification coaching program that included physical activity specific content relative to baseline levels of physical activity was effective in increasing the amount of physical activity as measured in weekly MET minutes with a median increase of 610.5 weekly MET minutes (range: −2,628 to 3,696) in the intervention group and 133.0 (range: −4976 to 25,638) in the control group with a significant between-group difference (*T* = 692.50; *P* = 0.002) [58]. An 8-week community-based stroke prevention program with a focus on increasing walking for exercise as one component of a secondary risk prevention program resulted in maintenance of the proportion of individuals that were participating in walking for exercise in the intervention group at 3 months (non-significant decline of 1.8%; *P* = 0.975), while the control group saw a significant decline of 16.9%; *P* = 0.021), resulting in a significant between-group difference at 3 months (*P* < 0.001) [59]. The study by Damush and colleagues [56] reported limited data about physical activity outcomes from their *‘Stroke Self-management Program’*. We were unable to obtain further data for analysis from the study’s authors. From the published results, there does appear to be an average increase of 47.6 min/week in self-reported time spent doing aerobic activity at 3 months in the intervention group and an average decline in the control group of 3 min/week [56]. However, these results should be interpreted with caution given the control group was more active than the intervention group at baseline (107.8 *vs*. 78.9 min/week on average, respectively). Gill and Sullivan’s *‘Stay Active and Stop Stroke’* program demonstrated limited benefits from this short intervention on the CABS-R Exercise subscale [57]. A significant increase in self-efficacy for exercise was seen in the intervention group at follow-up (*F*(1, 11) = 7.33; *P* = 0.02); however, this did not result in a significant difference between groups. In TBI, Brenner and colleagues reported limited data from the physical activity subscales HPLP-II Physical Activity Subscale and SRAHP Physical Activity and Exercise domain. Further data were unable to be obtained from the authors. The reported program outcomes showed no significant between-group differences [55].

#### Efficacy and acceptability of remote delivery

No study in this review utilized remote delivery of a self-management program in isolation from face-to-face delivery. Remote delivery via telephone was the predominant form of delivery in two studies [56,58]. Although efficacy of remote delivery in isolation cannot be fully established at this stage, current evidence does support the inclusion of remote delivery modes in self-management programs for individuals following stroke. Acceptability of delivery mode was not formerly assessed in either study. Attrition rates were low in both studies; however, reasons for attrition were not reported.

#### Program features associated with optimal clinical outcomes and client satisfaction

Due to the heterogeneity of outcome measures, as well as program content and delivery characteristics, a comparison of studies in order to determine features associated with best clinical outcomes is difficult. In addition, there was no analysis of client satisfaction in any of the studies included in this review. The amount of specific physical activity-related program content was not able to be clearly established in four of the five studies included in this review due to physical activity being a subtopic of a larger self-management program [55,56,58,59]. It was also difficult to establish the depth to which content was covered and the extent to which skills were taught and practiced. Education and goal setting were employed in all interventions and did not differentiate studies that obtained more positive results from those that demonstrated less efficacy of intervention. Sit and colleagues [59] were the only authors to implement the concept of individual preferences for both the choice of the risk behavior they wanted to focus on each week, as well as individual preferences for exercise pattern, duration, and pace. This was also the only study to focus on the formation of healthy habits as a part of their behavioral change intervention. Positive intervention results were also seen with the use of planning and scheduling [56,59] and coping strategies [56,58], while mixed success was seen with the implementation of barrier identification skills [55,57,58], problem solving [55-57], and self-monitoring [57-59].

## Discussion

This is the first review, to the authors’ knowledge, that has attempted to examine the clinical efficacy of self-management programs aimed at improving physical activity levels following ABI. This is an important contemporary issue in health care, and there is a growing body of literature in this area. However, there were a scant number of studies that met our stringent eligibility criteria. Many studies were excluded because they were not randomized or quasi-randomized controlled trials or because they did not utilize a self-management approach or examine physical activity specifically. An alternative approach to a future review in this field would be to include non-randomized studies and applying the GRADE approach to the examination of the quality of the evidence [43]. This may allow for more thorough examination of pragmatic trials conducted in this area.

The studies included in this review had a high risk of potential bias on many parameters. In part, this may be because some were smaller proof of concept studies, as is common in an emerging field. Nonetheless, the high risk of bias does limit interpretation of efficacy for the interventions investigated. With this taken into consideration, the results do show promising trends towards physical activity being enhanced through participation in a self-management program for individuals following stroke. This trend is not currently supported in TBI where the amount of research is even less, as highlighted by Pawlowski and colleagues. Their review of the status of physical activity research for individuals with TBI found only 6% (*n* = 4/63) of studies focused on the evaluation of behavior change intervention, and only 5% (*n* = 3/63) examined dissemination of health promotion programs [18]. More rigorous research is clearly needed in order to establish the efficacy and acceptability of self-management programs in improving physical activity levels for community-dwelling adults with ABI.

It was difficult to synthesize the results of the different self-management programs covered in this review, primarily because of variation in program content and delivery characteristics. Four studies examined self-management programs in which physical activity was only a small component of the overall program, rather than the main focus [55,56,58,59], making it difficult to establish the proportion and duration of the program that was focused on the acquisition of physical activity specific self-management skills in these studies. In the fifth study, although the focus of the program was specifically on physical activity [57], it was significantly shorter than the others at 1 h/week for 3 weeks in duration. In summary, while limited, the available evidence examined in this review indicates benefits in physical activity for stroke survivors of programs consisting of 6 to 8 sessions over 8 to 12 weeks. The evidence indicates that changing behavior related to physical activity is difficult in this population, particularly achieving sustained changes over time [65]. It is possible that too a short program does not allow for the establishment of skills needed for long-term behavior change to occur.

This review has demonstrated that self-management programs for stroke survivors that use a holistic, multifaceted approach offer some benefits in improving physical activity [56,58,59]. However, the concept of a self-management program that focuses on one risk factor, such as physical activity, also warrants further investigation. Sit and colleagues demonstrated positive results with a program that involved participants choosing the risk behavior on which they wanted to focus each week [59]. Such a focus on one self-selected risk factor has also been shown to have good effect on long-term physical activity levels in self-management programs with other populations, such as those with cardiovascular disease [66]. People with ABI often have complex disabilities and face multiple barriers and challenges in the self-management of physical activity. Therefore, a program that specifically targets physical activity may potentially be more effective in establishing long-term behavior change than a program that focuses on simultaneously changing numerous risk factors. Given the significance of physical inactivity to the global burden of chronic disease, this proposal warrants further investigation.

The professional background of the facilitators used in the programs reviewed here is also an issue of interest. The types of health professionals varied greatly between studies. Nurses were most commonly engaged as facilitators, with three of the five studies using at least one nurse in their facilitation team [55,56,58]. The experience and skills of the facilitators to help people increase physical activity following ABI is an important consideration in an analysis of efficacy of self-management programs to increase physical activity. However, this information was not reported in any of the included studies. People with ABI face many unique barriers to engaging in physical activity, such as mobility impairments, pain, fear, and limitations regarding access [4-8]. The experience of the facilitators in regard to changing physical activity behavior is an important factor to consider in any study that aims to increase physical activity levels of individuals with ABI.

The overall conclusions that can currently be drawn regarding efficacy of self-management programs for improving physical activity following ABI are limited. This is primarily because of the heterogeneity of methodological features such as the outcome measures used and how physical activity was operationalized. No study collected objective measures of physical activity such as from accelerometers or other devices. Although Sit and colleagues did have participants log data from pedometers for their own self-monitoring, these data were not reported in the study [59]. All five studies employed different self-report assessments of physical activity, each based on a different construct or aspect of physical activity. For example, one study measured minutes per week spent in aerobic activity [56] while another study examined attitudes and beliefs regarding exercise [57]. Additionally, in three of the five studies, the physical activity outcome was not the primary outcome [56,58,59]. In another study, the physical activity measure was a subscale of the primary outcome measure [55], which limits the power of the study to make conclusions about physical activity. Boger and colleagues have stated that the use of outcome measures which are related, indirect, or proxy indicators of self-management and that have questionable reliability and validity, contributes to an inability to sensitively evaluate the effectiveness of stroke self-management interventions [47]. Thus, in future research, employing objective measures of physical activity along with validated self-report measures that can capture participation in a broad range of physical activities is important and will enable a more rigorous investigation of the efficacy of self-management interventions aimed at in improving physical activity levels.

An additional limitation of this review may come from the common diversity seen in an ABI population. Studies examining both individuals with stroke and those with TBI were included in this review. There are obvious differences between these populations, for example, etiology and average age. There was also limited information regarding the specific mobility or physical activity status of the included participants. This may impact on both the examination of overall efficacy and the ability to translate these results into practice. However, all the participants were community-dwelling adults with the cognitive and communicative ability to participate in a self-management program.

A second objective of this review was to assess the effectiveness and acceptability of self-management programs delivered remotely, that is, via telephone, computer, posted workbooks, and the Internet. The evidence on this question is even more limited and preliminary. Two of the five studies utilized one form of remote delivery, specifically telephone, with both studies showing positive findings in terms of increasing physical activity [56,58]. As outlined above, ABI survivors face many barriers to participate in physical activity and difficulties in accessing self-management programs due to mobility impairments, transport limitations, lack of specialist resources, and cost [4-8]. Remote delivery of interventions may assist in overcoming some of these barriers and access issues [33]; however, research into this area is limited. Dishman and Buckworth conducted a meta-analysis of 127 studies examining the efficacy of interventions delivered via differing modes for increasing physical activity in community, worksite, school, home, and health-care settings. They reported that physical activity programs utilizing mediated delivery methods, such as the Internet, are more effective than those using just face-to-face methods [65]. Although this differs from the findings of Conn *et al*. [67], who found face-to-face delivery produced superior outcomes in healthy adults, a recent Cochrane review by Foster *et al*. [41] has shown consistent evidence to support the effectiveness of remote and web-based interventions for promoting physical activity in generally healthy community-dwelling adult populations. There are also promising results from a number of non-randomized stroke-specific studies utilizing telehealth interventions [68-71], and it is important to note that the acceptability of remote interventions may be examined in more detail in these earlier stage research studies. Further research into the efficacy of remote delivery of self-management programs for ABI survivors, specifically aimed at improving physical activity levels, is therefore warranted given the importance of physical activity and the difficulties of people with ABI report in accessing self-management programs.

This review also aimed to establish which features of self-management interventions were associated with the optimal clinical outcomes and client satisfaction for participants. Although specific content related to physical activity was difficult to quantify, a number of common features were seen across the five studies. For example, education and goal setting were features of all the intervention programs included in this review. This is important because improving health literacy through health education programs helps build the capacity of individuals to seek, access, comprehend, and effectively utilize health information and services [22,72,73]. Goal setting, when combined with improved health literacy, does appear to positively influence patients’ perceptions of self-care ability and engagement in rehabilitation following stroke [54]. However, education and goal setting did not differentiate a positive study from one that was less effective. It is difficult to establish whether programs were developed in a way that effectively targeted the level of health literacy of the users to allow for behavior change. Brenner and colleagues report on performing a pilot study of the program used in their study on eight participants with no resulting change to the materials [55], while Damush and colleagues report on developing their program with input from key stakeholders, including veterans with stroke [56]. Other authors report on building programs based on findings from local studies and focus groups [57,59]; however, although this may assist with content development, it does not guarantee that materials were targeted at the correct level of health literacy for users. The complexities of the manner in which these elements were delivered cannot be differentiated with the current evidence. Other program components were inconsistent between studies. These included self-monitoring, teaching coping strategies, planning and scheduling, barrier identification, problem solving, and habit formation.

Three of the five studies based their interventions on recognized psychological theories of behavior change, namely, the transtheoretical model, social cognitive model, and expanded health beliefs model [55-57]. Sit *et al*. did not specify a particular model of behavior change on which their program is based but do discuss the importance of modification of lifestyle habits as a component of behavioral change [59]. Kim and Kim also did not specify any theoretical basis to their interventions but utilized an educational framework with similar behavior change principles to the other studies examined [58]. Utilizing both psychological science and best educational practices can optimize the impact of self-management programs [22], and physical activity programs based on the principles of behavior modification have shown to be more effective than those based on cognitive modification [65,67]. A review of more than 550 pieces of high-quality research by de Silva [29] suggests that it is worthwhile to support self-management of individuals with chronic health conditions, particularly when there is a focus on behavior change and increasing self-efficacy, through approaches such as motivational interviewing and coaching with active goal setting. Future self-management programs aiming to increase physical activity following ABI should continue to adhere to these principles.

## Conclusions

The field of self-management of chronic health conditions is rapidly growing, and successes have been demonstrated in a range of conditions, such as depression and chronic pain. The application of this approach for individuals with ABI is emerging. To date, there are a limited number of trials that have specifically investigated the efficacy of self-management to improve physical activity in this population. However, the risk of bias of these studies is generally high, and analysis is limited by heterogeneity in study interventions, methodology, measures, and diversity of the ABI population. Based on the results of this review, the efficacy of self-management programs in increasing physical activity levels in community-dwelling adults following ABI is still unknown. Moreover, the efficacy and acceptability of remotely delivered self-management programs for increasing physical activity levels after ABI is also unknown.

Further research into physical activity following self-management interventions for community-dwelling adults with ABI is required in order to properly establish efficacy and implications for practice. This research should be designed, undertaken, and reported on in a manner that reduces the potential for bias and allows for establishment of efficacy. Remote delivery methods also warrant further research given the potential they offer in regard to improving access, overcoming barriers, and changing health behaviors.

## Additional file

Additional file 1: **MEDLINE search strategy.** Final search strategy used in MEDLINE.

## Abbreviations

ABI: Acquired brain injury
CABS-R: Cerebrovascular attitudes and beliefs scale - revised
CENTRAL: Cochrane central register of controlled trials
eHBM: expanded health beliefs model
FASD: Fetal alcohol spectrum disorder
HPLP-II: Health promoting lifestyle practices - II
IPAQ: International physical activity questionnaire
MET: Metabolic equivalent of task
PRISMA: Preferred reporting items for systematic reviews and meta-analyses
SCI-EXPANDED: Science citation index expanded
SCT: Social cognitive theory
SRAHP: Self-rated abilities for health practices
TBI: Traumatic brain injury
TIA: Transient ischaemic attack
TTM: Transtheoretical model
WHO: World health organization
