# Supplementary material for: A systematic review of the efficacy of self-management programs for increasing physical activity in community-dwelling adults with acquired brain injury (ABI)
Source: Syst Rev. 2015 Apr 19;4:51. doi: 10.1186/s13643-015-0039-x (PMC4422226; doi:10.1186/s13643-015-0039-x)
Supplement: Additional file 1: — MEDLINE search strategy. Final search strategy used in MEDLINE. [file 13643_2015_39_MOESM1_ESM.pdf]

The efficacy of self-management programs for increasing physical activity in community-dwelling adults with acquired brain injury (ABI): A systematic review.

Medline search strategy

1. exp Self Care/
2. exp health education/ or exp patient education as topic/
3. exp consumer participation/ or exp patient participation/
4. exp health communication/ or exp health promotion/
5. exp Self Concept/ or exp Self Efficacy/
6. (self adj care*).mp.
7. (self adj manage*).mp.
8. (patient adj educat*).mp.
9. (self adj monitor*).mp.
10. (self adj efficacy).mp.
11. (self adj concept).mp.
12. ((consumer or patient) adj participat*).mp.
13. ((consumer or patient) adj inform*).mp.
14. (health adj educat*).mp.
15. (health adj promot*).mp.
16. 1 or 2 or 3 or 4 or 5 or 6 or 7 or 8 or 9 or 10 or 11 or 12 or 13 or 14 or 15
17. exp Motor Activity/
18. exp "activities of daily living"/ or exp leisure activities/ or exp recreation/
19. exp gait/ or exp locomotion/ or exp walking/
20. exp sports/ or exp physical fitness/
21. exp Exercise/ or exp Exercise Therapy/
22. exp Health Behavior/
23. (physical adj activity).mp.
24. (leisure or recreation*).mp.
25. (sport* or fit* or exercis*).mp.
26. (walk* or ambulat* or mobil* or locomotion or gait).mp.
27. 17 or 18 or 19 or 20 or 21 or 22 or 23 or 24 or 25 or 26
28. 16 and 27
29. exp brain damage, chronic/ or exp brain injuries/ or exp cerebrovascular disorders/
30. (brain adj (injur* or damage)).mp.
31. stroke*.mp.
32. (cerebrovascular adj accident*).mp.
33. exp Stroke/
34. 29 or 30 or 31 or 32 or 33
35. 28 and 34
36. Randomized controlled trial.pt.
37. random*.mp.
38. trial*.mp.
39. control*.mp.
40. controlled clinical trial.pt.
41. placebo*.mp.
42. (intervention adj group*).mp.
43. (treatment adj group*).mp.
44. 36 or 37 or 38 or 39 or 40 or 41 or 42 or 43
45. 35 and 44
46. limit 45 to humans
47. remove duplicates from 46
